# Augmenting cancer registry data with health survey data with no cases in common: the relationship between pre-diagnosis health behaviour and post-diagnosis survival in oesophageal cancer

**DOI:** 10.1186/s12885-020-06990-3

**Published:** 2020-06-01

**Authors:** Paul P. Fahey, Andrew Page, Glenn Stone, Thomas Astell-Burt

**Affiliations:** 1 School of Science and Health, Western Sydney University, Locked Bag 1797, Penrith, NSW 2751 Australia; 2 Translational Health Research Institute, Western Sydney University, Locked Bag 1797, Penrith, NSW 2751 Australia; 3 School of Computing, Engineering and Mathematics, Western Sydney University, Locked Bag 1797, Penrith, NSW 2751 Australia; 4 Population Wellbeing and Environment Research Lab (PowerLab), School of Health and Society, Faculty of Social Sciences, University of Wollongong, Wollongong, NSW 2522 Australia

**Keywords:** Cancer registries, Alcohol drinking, Oesophageal neoplasms, Exercise, Obesity, Tobacco smoking

## Abstract

**Background:**

For epidemiological research, cancer registry datasets often need to be augmented with additional data. Data linkage is not feasible when there are no cases in common between data sets. We present a novel approach to augmenting cancer registry data by imputing pre-diagnosis health behaviour and estimating its relationship with post-diagnosis survival time.

**Methods:**

Six measures of pre-diagnosis health behaviours (focussing on tobacco smoking, ‘at risk’ alcohol consumption, overweight and exercise) were imputed for 28,000 cancer registry data records of US oesophageal cancers using cold deck imputation from an unrelated health behaviour dataset. Each data point was imputed twice. This calibration allowed us to estimate the misclassification rate. We applied statistical correction for the misclassification to estimate the relative risk of dying within 1 year of diagnosis for each of the imputed behaviour variables. Subgroup analyses were conducted for adenocarcinoma and squamous cell carcinoma separately.

**Results:**

Simulated survival data confirmed that accurate estimates of true relative risks could be retrieved for health behaviours with greater than 5% prevalence, although confidence intervals were wide. Applied to real datasets, the estimated relative risks were largely consistent with current knowledge. For example, tobacco smoking status 5 years prior to diagnosis was associated with an increased age-adjusted risk of all cause death within 1 year of diagnosis for oesophageal squamous cell carcinoma (RR = 1.99 95% CI 1.24,3.12) but not oesophageal adenocarcinoma RR = 1.61, 95% CI 0.79,2.57).

**Conclusions:**

We have demonstrated a novel imputation-based algorithm for augmenting cancer registry data for epidemiological research which can be used when there are no cases in common between data sets. The algorithm allows investigation of research questions which could not be addressed through direct data linkage.

## Background

In 2011 it was estimated that that the cost of maintaining the United States’ National Program of Cancer Registries was $US60.77 per case [1]. The estimated number of new United States cancer cases in 1999 was 1,291,451 [2] and 1,762,450 in 2019 [3] an increase of 36% in 20 years. As in any public investment, there is always a need to maintain, and indeed increase, benefits of cancer registries relative to costs.

The role of cancer registries has changed considerably over time [4]. Since the 1990s, for example, the development of specialised data linkage infrastructure has open wide new research applications [4]. However, data linkage may not be feasible in all circumstances. There are still research questions which are waiting for a suitable method of analysis.

Oesophageal cancer is the seventh most common cancer by site [5], has low survival [6], and caused an estimated 1 in 20 cancer deaths worldwide in 2018 [5]. It has been estimated that 71% of male and 59% of female oesophageal cancer deaths in the US arise from modifiable health behaviours: including smoking (50%), alcohol consumption (17%) and excess body weight (27%) [7]. The impact of pre-diagnosis health behaviour on oesophageal cancer survival is uncertain. As survival times are short, the carry-over effect of pre-diagnosis behaviour may be important, and potentially impact treatment choices [8]. Further, as health behaviours in populations change over time [9, 10], predicting the impact of behaviour on cancer survival would assist in forecasting future disease burden and health service requirements.

Associations between oesophageal cancer incidence and health behaviour (including tobacco smoking, alcohol consumption, body mass index and physical activity) differ by histological sub-type [11, 12] with oesophageal squamous cell carcinoma (ESCC) and oesophageal adenocarcinoma (EAC) usually examined separately. Similar differences may exist for survival time [13, 14].

Nowadays, cancer survival data is generally available through cancer registries [15], but not data on pre-diagnosis health behaviour. Registry data needs to be augmented with additional data collection or linkage to external data sources. Additional data collection can be time consuming, expensive and subject to survivor bias [16] and data linkage needs the same individuals to be present and identifiable in both data collections and is less feasible for rare disease like oesophageal cancer.

When faced with missing data, researchers sometimes use imputation [17]. Imputing data is likely to lead to misclassification of health behaviours (such as smokers classified as non-smokers and vice-versa). However, repeated observations of the same behaviour can be used to quantify, and subsequently correct for misclassification [18]. In this paper we investigate the possibility that, with large datasets and careful calibration, imputing a completely missing variable could return valid results. We describe and evaluate an algorithm for assessing the relationship between pre-diagnosis health behaviours and survival at one-year post-diagnosis for oesophageal cancer where survival is derived from cancer registry data and key health behaviours are fully imputed using unrelated health survey data.

## Methods

### Data sources

Oesophageal cancer cases were extracted from the Surveillance, Epidemiology, and End Results Program (SEER) cancer registries database, which combines data from cancer registries in up to 13 US States covering up to 28% of the US population [19]. Available data included patient demographics and outcomes (including survival time).

All records of primary oesophageal cancers diagnosed between 2006 to 2014 were downloaded using the SEER*Stat utility [20]. After excluding 112 cases < 35 years of age as atypical, the dataset contained 34,972 oesophageal cancer cases.

Health behaviour data of US residents were extracted from the Behavioural Risk Factor Surveillance System (BRFSS) [21]. This telephone survey of the adult population of US residents (all States) has been conducted annually since 1984. All 3,018,830 records from 2001 to 2009 were included.

Given that health behaviour can change after diagnosis [22, 23] the BRFSS health behaviour best represented the health behaviour of oesophageal cancer cases pre-diagnosis. We added a 5-year lag to minimise the risk of early symptoms influencing behaviour. The initial year was the earliest year in which BRFSS used a consistent definition for health behaviours selected for the present study. The end year was the most recently available SEER cancer registry data which allowed at least 12-months follow-up.

### Outcomes, predictors and subgroups

The dichotomous outcome was all-cause mortality within 1 year of diagnosis.

Six self-reported measures of health behaviour were selected based on previous associations with oesophageal cancer [11, 24] and availability in the BRFSS dataset:

- Current tobacco smoking (yes or no), defined as daily or less than daily smoking;
- Alcohol consumption – possible binge drinking (yes or no), defined as ≥5 standard drinks for males or ≥ 4 standard drinks for females on at least one occasion in the month prior to survey;
- Alcohol consumption – possible heavy drinking (yes or no), defined as > 2 standard drinks per day for men and > 1 standard drink per day for women in the month prior to survey;
- Physical activity (yes or no), defined as any physical activity or exercise in the past 30 days other than for regular job;
- Obese (yes/no), defined as body mass index ≥30 kg/m^2^; and
- Current tobacco smoking with regular alcohol (yes or no), defined as current tobacco smoking with ≥1 standard drink of alcohol per day on average in the previous month.

Histological subgroups were defined using International Classification of Diseases for Oncology, third edition (ICD-O-3) with 805–808 indicating ESCC (*n* = 10,454) and 814–838 indicating EAC (*n* = 17,950).

### Imputation method and covariates

The complete absence of data on health behaviour meant that regression-based imputation and multiple imputation could not be used [25]. Random cold deck imputation [17] based on demographic strata was appropriate, as there were demographic variables in common between the two datasets and individuals from the same demographic group have a greater likelihood of engaging in similar health behaviours [26].

In random cold deck imputation individuals are allocated into strata according to auxiliary variables and then, within each stratum, one ‘donor’ record is randomly selected for each ‘recipient’ record. The BRFSS health behaviour data were the donor records and the SEER cancer registry data were the recipients. The recipient record is assigned the behaviour of the donor record. The more, and the more informative, the auxiliary variables the greater the chance the imputed behaviour will be correct.

Six auxiliary variables were used:

- Age category at diagnosis (5-year groups from 35-39y to 75-79y then >80y);
- Gender (male; female);
- Marital status (married, including common law; single or never married; widowed; divorced);
- Race (white; black; Asian or Pacific Islander; American Indian or Alaska Native);
- State of residence (Alaska; California; Connecticut; Georgia; Hawaii; Iowa; Kentucky; Louisiana; Michigan; New Jersey; New Mexico; Utah; Washington);
- Year of diagnosis (2006 to 2014).

To produce the 5-year lag, we defined the donor records to be BRFSS health behaviour records which were 5 years earlier and one age-group younger than the corresponding SEER cancer case.

There were 37,440 possible combinations of the auxiliary variable categories, 7397 of which occurred within the SEER oesophageal cancer cases. Of these, 6986 (94.4%) contained at least one eligible BRFSS donor record.

To allow calibration, we randomly selected two BRFSS donor records for each SEER case (without replacement), such that each cancer case had two imputed values for each lifestyle variable. Where donor records were exhausted before cancer cases, the cancer case was omitted from the analysis (see Additional file 1).

### Missing data, exclusions and the final dataset

Approximately 80% of the 35,084 eligible oesophageal cancer cases were included in the analyses. (Additional file 2). SEER cases were excluded for missing survival time or auxiliary variables (*n* = 2784, 8.0%) or failing to find two donor records (from 4353 to 4453 (12.4 to 12.7%) varying between health behaviours). Cases without two donor records were more likely to be older, from earlier study years and California residents (Additional file 3).

Only 458,780 of the BRFSS health behaviour records matched the SEER cases on the auxiliary variables. The number with missing health behaviour ranged from 564 (0.1%) for physical activity to 17,624 (3.9%) for obesity. To avoid imputing a missing value into a missing value, these records were excluded. To avoid cumulative effects, we created six separate donor datasets (each containing complete cases for one of the six health behaviours) and imputed each health behaviour independently.

### Calibrating the effectiveness of imputation

We used the paired imputed values to calibrate the imputation process (see Additional file 4). In brief, let *p*_*i*_ represent the proportion of imputed values where the behaviour is present. If the imputation process retained no information on behaviour, the expected proportion of behaviour present to behaviour present matches is ${p}_i^2$ - the agreement arising through chance alone. If the imputation process is informative, the proportion of behaviour present to behaviour present matches is greater than chance. We modelled these excess matches as *p*_*i*_(1 − *p*_*i*_) *ρ* where *ρ* is a measure of correlation [27].

We estimated *p*_*i*_ as the proportion imputed to have the behaviour (averaged across the two imputed values) and estimated *ρ* using the phi coefficient (the correlation coefficient for dichotomous variables) between the pairs of imputed values. All analyses were conducted separately for each health behaviour.

### Statistical analysis

For each behaviour, we cross-tabulated the first set of imputed values against 1 year survival status and calculated the relative risk of death within 1 year, *RR*_*i*_. The subscript *i* signifies that the imputed data were used in the calculations.

Other potential predictors of survival times were investigated using log-binary regression with associated log likelihood ratio statistics and area under the receiver operator curves (Additional file 5). Age was identified as a confounder as both post-diagnosis survival and proportion recording each health behaviours were lower among older age groups (Additional file 5). To adjust for this, age-adjusted relative risks, *adjRR*_*i*_, were estimated using the Cochrane-Mantel-Haenzel method [28]. Other potential demographic predictors of survival were found to be of lesser impact or confounded with age (see Additional file 5).

Beyond the demographic variables, cancer stage at diagnosis (coded by SEER according to the AJCC Cancer Staging Manual 6th Edition [29]) was confirmed as a stronger predictor of survival (Additional file 5) but, occurring after health behaviour exposure, may partially lie on the disease pathway. That is, smokers may have more advanced disease at diagnosis due to their smoking and so correcting for cancer stage at diagnosis may falsely attenuate the association between pre-diagnosis smoking and survival post diagnosis [30]. Subgroup analyses for cancer stage at diagnosis are provided in Additional file 8.

Non-differential misclassification errors will, barring random error and confounding, attenuate the estimated relative risk toward the null [31]. The mathematical relationship between the relative risk using the imputed data, *RR*_*i*_, and the true relative risk for the cancer cases, *RR*_*T*_, is derived in Additional file 6. In brief, if the prevalence of behaviour is the same between the donor records and cancer cases in each stratum, the true relative risk can be estimated using

$$ {RR}_T=1-\frac{\left({RR}_i-1\right)}{\left({RR}_i-1\right){p}_i\left(\ 1-\rho \right)-\rho } $$

Extreme values of *p*_*i*_ and/or *ρ* can be problematic. For example, when *ρ* = 0, *RR*_*T*_ is negative: an impossible value for a relative risk.

Random cold deck imputation was repeated 100 times, separately for each of the six health behaviours. As donor records were selected at random within strata, each statistic varied between repetitions. Results were reported as the median value from the 100 repetitions with the associated 2.5 and 97.5 percentiles as empirical 95% confidence intervals. We report subgroup analyses for ESCC and EAC. Where more than 5% of the estimates of the true relative risk *RR*_*T*_ were impossible, the imputation process was labelled as ‘failed’.

### Checking the algorithm with simulated data

In the absence of a cohort showing the true relationship between pre-diagnosis health behaviour and post-diagnosis survival time, we used simulated data to test the algorithm.

The first set of imputed behaviour was designated to be the ‘true’ health behaviour of each cancer case. For each health behaviour we separately simulated seven survival status variables (repeated 100 times): to produce relative risks of 0.50, 0.66, 0.80, 1.00, 1.25, 1.50 and 2.00 while maintaining the overall rate of the health behaviour *p*_*i*_ and 1 year death rate (Additional file 7).

The imputed relative risks were obtained using the second set of imputed health behaviours. As the second set of imputed values were selected independently and without replacement, they had a similar relationship with the first set of simulated data as with the actual cancer cases. The main difference is that the simulated survival data, being based only on the behaviour of interest, have no relationship with (confounding from) any other variables. The true data were likely to display more complex relationships.

## Results

### Calibrating the imputation

The estimated proportion of cancer cases with a given health behaviour, *p*_*i*_, ranged from a median of 0.737 for physical activity to 0.034 for current smoking with regular drinking (Table 1). The phi coefficients, *φ*, show that there is usually a positive correlation between the two imputed values, albeit weak (medians between 0.008 and 0.077). This confirms that some information about health behaviour is being conveyed through the random cold deck imputation. The value *np*_*i*_(1 − *p*_*i*_)*ρ*, the number of correct matches greater than would be expected through chance, quantifies the information conveyed through the imputation. ‘Heavy drinking’, and ‘current smoking with regular drinking’, had the lowest prevalence (median of 0.05 or less), the lowest correlations between imputed observations (median less than 0.025) and hence lowest information (medians below 20 matches beyond chance).

**Table 1:** The estimated proportions with each health behaviour, the phi coefficient between imputed values and the estimated excess matches for each analysis

Behaviour 5 years before diagnosis	N	Estimated proportion with behaviour, $\hat{p_i}$		Estimated phi coefficient, $\hat{\rho}$ = *φ*		Estimated excess matches, $n\hat{p_i}\left(1-\hat{p_i}\right)\hat{\rho}$	
		Median	95% CI	Median	95% CI	Median	95% CI
Current smoking							
overall	27,835	0.159	0.157,0.162	0.071	0.059,0.084	262.2	220.1312.2
ESCC	8914	0.166	0.162,0.170	0.077	0.061,0.097	94.8	74.5120.7
EAC	15,726	0.157	0.153,0.159	0.066	0.052,0.081	137.0	107.4169.5
Binge drinking							
Overall	27,750	0.100	0.098, 0.102	0.060	0.049,0.077	150.5	121.5192.1
ESCC	8891	0.086	0.082,0.089	0.060	0.042,0.086	42.2	29.8,61.1
EAC	15,673	0.109	0.106,0.111	0.058	0.042,0.079	88.6	63.6120.3
Heavy drinking							
Overall	27,749	0.048	0.047,0.050	0.011	0.002,0.025	14.3	2.7,32.0
ESCC	8888	0.046	0.043,0.049	0.015	−0.002,0.036	5.7	− 0.7,14.2
EAC	15,676	0.050	0.048,0.052	0.008	−0.004,0.028	6.0	−3.0,20.8
Physical activity							
Overall	27,830	0.737	0.734,0.740	0.034	0.026,0.046	185.1	139.4247.4
ESCC	8912	0.716	0.709,0.721	0.036	0.016,0.056	64.7	29.6100.2
EAC	15,724	0.750	0.746,0.754	0.031	0.013,0.047	91.4	40.0,138.4
Obese							
Overall	27,796	0.257	0.254,0.261	0.030	0.020,0.042	160.2	108.4226.8
ESCC	8898	0.262	0.255,0.268	0.045	0.024,0.061	77.0	41.4104.6
EAC	15,709	0.256	0.251,0.261	0.023	0.012,0.041	67.8	35.0,122.4
Current smoking with regular drinking							
Overall	27,735	0.034	0.033,0.035	0.022	0.009,0.038	19.8	8.0,34.2
ESCC	8883	0.031	0.029,0.033	0.024	−0.000,0.049	6.2	−0.0,13.5
EAC	15,670	0.035	0.034,0.037	0.021	0.004,0.042	11.5	2.1,22.4

$\hat{p_i}$ proportion of imputed values where the health behaviour is present

$\hat{\rho}$ = *φ* the correlation between the pairs of imputed values (calculated as the phi coefficient)

$n\hat{p_i}\left(1-\hat{p_i}\right)\hat{\rho}$= the excess number of correct matches greater than would be expected through chance alone

*Median* median of 100 repetitions of the imputation algorithm,

95% CI = empirical 95% confidence interval created from the 2.5 and 97.5 percentiles obtained from 100 repetitions of the imputation algorithm,

*N* number of SEER oesophageal cancer cases receiving data from two donor records from the BRFSS health behaviour datasets

*ESCC* oesophageal squamous cell carcinoma,

*EAC* oesophageal adenocarcinoma

### Analyses using simulated survival status

The simulated relative risks of survival were accurate to two-decimal places and precise (with a maximum margin of error of 0.07) (Table 2). The relative risks obtained by using the (second) imputed behaviour (*RR*_*i*_) were substantially attenuated toward the null differing from 1.0 only in the second decimal place.

**Table 2:** Result of simulation-based testing of whether or not the imputation can be used to predict relative risk

Target RR	Simulated data RR		Imputed RR (*RR*_*i*_)		Impossible Result (*RR*_*T*_ < 0)	Estimated true RR (*RR*_*T*_)	
	Median	95% CI	Median	95% CI	Frequency	Median	95% CI^b^
Current smoking							
RR = 0.5	0.501	0.475,0.521	0.964	0.934,0.993^a^	0	0.519	0.163,0.904^a^
RR = 0.66	0.660	0.635,0.683	0.973	0.944,0.999^a^	0	0.638	0.300,0.985^a^
RR = 0.80	0.799	0.771,0.823	0.983	0.952,1.017	0	0.753	0.375,1.226
RR = 1.00	1.001	0.976,1.026	0.997	0.967,1.027	0	0.957	0.577,1.444
RR = 1.25	1.249	1.220,1.287	1.017	0.989,1.048	0	1.254	0.856,1.793
RR = 1.50	1.499	1.465,1.528	1.032	1.005,1.059^a^	0	1.486	1.069,1.947^a^
RR = 2.00	2.000	1.974,2.034	1.064	1.034,1.092^a^	0	2.047	1.542,2.532^a^
Binge drinking							
RR = 0.5	0.501	0.474,0.526	0.967	0.940,0.996^a^	0	0.478	0.087,0.927^a^
RR = 0.66	0.659	0.624,0.692	0.976	0.945,1.015	1	0.629	0.173,1.316
RR = 0.80	0.798	0.758,0.830	0.988	0.959,1.025	0	0.805	0.341,1.448
RR = 1.00	0.997	0.963,1.033	0.999	0.971,1.032	0	0.981	0.518,1.492
RR = 1.25	1.245	1.213,1.278	1.016	0.984,1.054	0	1.271	0.739,2.029
RR = 1.50	1.499	1.463,1.534	1.030	0.990,1.068	0	1.517	0.831,2.246
RR = 2.00	1.999	1.978,2.028	1.058	1.021,1.093^a^	0	2.014	1.352,2.717^a^
Heavy Drinking							
RR = 0.5	0.500	0.450,0.548	0.995	0.945,1.046	40	failed	failed
RR = 0.66	0.661	0.606,0.697	0.995	0.946,1.046	34	failed	failed
RR = 0.80	0.799	0.746,0.847	0.997	0.944,1.053	43	failed	failed
RR = 1.00	0.997	0.949,1.045	0.998	0.940,1.041	32	failed	failed
RR = 1.25	1.251	1.210,1.300	1.003	0.959,1.053	22	failed	failed
RR = 1.50	1.497	1.459,1.535	1.012	0.956,1.059	24	failed	failed
RR = 2.00	Not possible	Not possible					
Physical activity							
RR = 0.5	0.500	0.491,0.509	0.974	0.951,0.997^a^	0	0.504	0.319,0.901^a^
RR = 0.66	0.659	0.645,0.671	0.983	0.959,1.006	0	0.632	0.367,1.231
RR = 0.80	0.800	0.782,0.818	0.993	0.971,1.017	0	0.833	0.449,1.907
RR = 1.00	1.002	0.976,1.022	1.001	0.978,1.021	0	1.025	0.488,2.092
RR = 1.25	1.250	1.219,1.276	1.006	0.977,1.030	0	1.206	0.541,2.961
RR = 1.50	1.499	1.455,1.549	1.013	0.987,1.037	2	1.514	0.722,4.078
RR = 2.00	2.003	1.939,2.083	1.021	1.002,1.047*	3	2.127	1.055,10.987^a^
Obese							
RR = 0.5	0.499	0.485,0.517	0.983	0.960,1.008	1	0.550	0.028,1.322
RR = 0.66	0.660	0.634,0.680	0.989	0.962,1.016	2	0.665	0.114,1.772
RR = 0.80	0.802	0.777,0.823	0.995	0.967,1.015	1	0.846	0.316,1.676
RR = 1.00	1.002	0.981,1.024	0.999	0.980,1.024	0	0.962	0.461,2.067
RR = 1.25	1.250	1.222,1.274	1.009	0.989,1.030	0	1.335	0.601,2.300
RR = 1.50	1.500	1.468,1.534	1.014	0.987,1.039	0	1.440	0.606,2.796
RR = 2.00	2.002	1.961,2.041	1.025	0.997,1.044	0	1.995	0.886,3.234
Current smoking with regular drinking							
RR = 0.5	0.504	0.441,0.550	0.988	0.931,1.034	37	failed	failed
RR = 0.66	0.660	0.600,0.713	0.997	0.932,1.066	31	failed	failed
RR = 0.80	0.797	0.744,0.863	0.991	0.928,1.052	34	failed	failed
RR = 1.00	0.996	0.943,1.049	1.001	0.940,1.059	25	failed	failed
RR = 1.25	1.250	1.183,1.298	1.009	0.954,1.059	16	failed	failed
RR = 1.50	1.497	1.454,1.545	1.000	0.958,1.065	19	failed	failed
RR = 2.00	Not possible	Not possible					

Target RR – the relative risk we attempted to achieve in the simulated data

Simulated data RR – the relative risk which was actually achieved between the first imputed value and the simulated one-year survival status

Imputed RR (*RR*_*i*_) – the relative risk calculated using the second imputed data point as the imputed behaviour

Impossible result – instances where the estimated true relative risk was impossible (a negative value)

Estimated True RR (*RR*_*T*_) – the estimated true relative risk derived from the imputed relative risk and calibration parameters $\hat{p_i}$ and $\hat{\rho}$

*Median* median of 100 repetitions of the imputation algorithm,

95% CI = empirical 95% confidence interval created from the 2.5 and 97.5 percentiles obtained from 100 repetitions of the imputation algorithm,

^a^ 95% confidence intervals exclude no association (i.e. exclude relative risk equals 1)

^b^ excludes impossible result

Estimation of the true relative risk from the imputed relative risk failed for the two least common health behaviours: ‘heavy drinking’ and ‘current smoking with regular drinking’. For the other four behaviours, the median of the estimated true relative risk was accurate to one, and often two, decimal places. However, the confidence intervals were wide and few excluded no association.

### Analyses using true survival status

When imputing the health behaviours onto SEER cancer cases, the median imputed relative risks (*RR*_*i*_) are attenuated to close to 1.0 (Table 3). Less expectedly, most of the median risks are less than 1.0; suggesting that most behaviours were associated with a lower rate of death within one year of diagnosis. Many of the age-adjusted imputed relative risks had the opposite direction of association confirming the potential for confounding by age. Current tobacco smoking 5 years prior to diagnosis was detrimental to one-year survival after diagnosis following adjustment for age, particularly in ESCC where the estimated relative risk was 2.0 (95%CI 1.24, 3.12). For ESCC, the median relative risk for binge drinking 5 years prior to diagnosis was 1.52 although the range of possible relative risks was wide (95% CI 0.44,2.75). Similar results were seen for obesity (ESCC estimated RR 1.73, 95%CI 0.83,4.17). Physical activity 5-years prior to diagnosis was protective for survival with median estimated relative risks of approximately 0.50 (95%CI 0.31, 1.03) for oesophageal cancer overall.

**Table 3:** Estimated relative risks of 1-year survival derived from imputed pre-diagnosis behaviours for SEER oesophageal cancer cases, 2006–2014; unadjusted and age adjusted

	Imputed RR (*RR*_*i*_)		Impossible Result (*RR*_*i*_ < 0)	Estimated True RR (*RR*_*T*_)		Age-adjusted Imputed RR (*adjRR*_*i*_)		Impossible Result (*adjRR*_*i*_ < 0)	Age-adjusted Estimated True RR (*adjRR*_*T*_)	
	Median	95% CI	Frequency	Median	95% CI	Median	95% CI	Frequency	Median	95% CI
Current smoking										
All	0.986	0.954,1.009	0	0.806	0.380,1.130	1.051	1.014,1.078	0	1.794	1.215,2.357^a^
ESCC	1.025	0.981,1.067	0	1.349	0.733,2.142	1.064	1.016,1.111	0	1.990	1.240,3.117 ^a^
EAC	0.959	0.914,1.000 ^a^	5	0.478	0.039,1.003 ^b^	1.038	0.985,1.085	0	1.613	0.785,2.571
Binge drinking										
All	0.933	0.900,0.964	49	failed	failed	0.997	0.961,1.032	1	0.951	0.445,1.539 ^b^
ESCC	0.998	0.936,1.059	4	0.991	0.167,1.995 ^b^	1.033	0.968,1.101	0	1.515	0.440,2.754
EAC	0.914	0.863,0.961 ^a^	72	failed	failed	0.989	0.935,1.046	3	0.818	0.181,1.890 ^b^
Heavy drinking										
All	0.981	0.932,1.028	61	failed	failed	1.010	0.963,1.060	23	failed	failed
ESCC	0.995	0.912,1.066	48	failed	failed	1.012	0.929,1.088	36	failed	failed
EAC	0.974	0.907,1.039	66	failed	failed	1.011	0.938,1.077	35	failed	failed
Physical activity										
All	0.954	0.934,0.978 ^a^	0	0.319	0.165,0.564 ^a^	0.974	0.956,1.001	0	0.507	0.307,1.030
ESCC	0.959	0.925,0.991	2	0.345	0.073,0.811 ^a,b^	0.971	0.933,1.003	1	0.452	0.102,1.071^b^
EAC	0.957	0.929,0.986 ^a^	1	0.311	0.109,0.675 ^a,b^	0.984	0.954,1.013	0	0.627	0.285,2.180
Obese										
All	0.969	0.946,0.993 ^a^	24	failed	failed	1.008	0.983,1.036	0	1.262	0.559,2.931
ESCC	1.000	0.968,1.039	0	1.004	0.134,2.378	1.027	0.992,1.068	0	1.733	0.834,4.167
EAC	0.949	0.917,0.987 ^a^	76	failed	failed	0.996	0.960,1.035	8	failed	failed
Current smoking with regular drinking										
All	0.987	0.930,1.058	40	failed	failed	1.044	0.986,1.120	2	3.254	0.771,11.843 ^b^
ESCC	1.044	0.946,1.146	12	failed	failed	1.076	0.973,1.180	11	failed	failed
EAC	0.963	0.861,1.052	60	failed	failed	1.032	0.919,1.123	13	failed	failed

Imputed RR (*RR*_*i*_) – the relative risk calculated using the imputed behaviour

Impossible result – instances where the estimated true relative risk was impossible (a negative value)

Estimated True RR (*RR*_*T*_) – the estimated true relative risk derived from the imputed relative risk and calibration parameters $\hat{p_i}$ and $\hat{\rho}$

*Median* median of 100 repetitions of the imputation algorithm,

95% CI = empirical 95% confidence interval created from the 2.5 and 97.5 percentiles obtained from 100 repetitions of the imputation algorithm,

^a^ 95% confidence intervals exclude no association (i.e. exclude relative risk equals 1)

^b^ excludes impossible result

Estimates of the relative risks could not be retrieved for the less common behaviours ‘heavy drinking’ and ‘current smoking with regular drinking’. The one relative risk which was retrieved - a median RR of 3.35 for current smoking with regular drinking in all oesophageal cancer - was accompanied by wide uncertainty (95% CI 0.77,11.84).

Subgroup analyses on cancer stage at diagnosis (Additional file 8), suggests that pre-diagnosis health behaviours have stronger relationships with one-year survival in those who are not metastatic at diagnosis.

## Discussion

This study shows that an entirely missing variable can be imputed and return accurate estimates of relative risks. Nearly all correlation coefficients were positive, indicating that the imputation conveyed some information about health behaviour, although confidence intervals were wide. However, for the less common behaviours (heavy drinking and current smoking with regular drinking), no interpretable information could be retrieved.

The choice of health behaviour variables was restricted to measures available through the BRFSS health survey. However, the results are consistent with the literature. We found that tobacco smoking 5 years prior to diagnosis was associated with increased risk of death 1 year after diagnosis in ESCC (RR = 1.99, 95% CI 1.24,3.12) and, with less certainty, EAC (RR = 1.61, 95% CI 0.79,2.57). Recent meta analyses estimated hazard ratios (HRs) of 1.41 (95% CI 1.22,1.64) and 1.41 (95% CI 0.96,2.09) for current smoking relative to never smoked in mainly ESCC populations [32, 33] and 1.19 (95% CI 1.04,1.36) for ever smoking compared to never smoked in ESCC [24] with no evidence of association between smoking and survival in EAC [24, 33]. The unadjusted protective effects of smoking has also been reported [34, 35] as has the change in the direction of the association following age adjustment [35].

A previous meta-analysis found that ever drinking alcohol had a detrimental association with survival in ESCC (HR 1.36, 95% CI 1.15, 1.61) but not in EAC (HR = 1.08 95% CI 0.85, 1.37) [24]. More recent results from China (HR = 1.58, 95% CI 1.21,2.07 [36, 37], HR = 1.45 95% CI 1.13,1.87 [37]) and Japan (HR = 2.37 95% CI 1.24,4.53 [38]) also support the detrimental impact of pre-diagnosis alcohol consumption on survival in ESCC. We could not estimate the association between heavy drinking and survival. However, for binge drinking five years prior to diagnosis, the median relative risk was 1.52 in ESCC, although the confidence interval (95% CI 0.44,2.75) allows no association.

Previous studies have reported that pre-diagnosis smoking with regular alcohol consumption produced a disproportionately high risk to post-diagnosis survival in ESCC (HR 3.84, 95% CI 2.02,7.32 [13]). We observed a similar association (RR = 3.25, 95% CI 0.77,11.84) with wider confidence intervals.

In relation to obesity, a recent North American study [39] found self-reported obesity was associated with lower survival times in EAC compared to normal weight (HR 1.77, 95% CI 1.25, 2.51) and a 27 year follow-up of 29,446 participants in China [40] found higher body mass index protective of death from ESCC (HR = 0.97 per unit increase, 95% CI 0.95,0.99). We found, in contrast, that obesity 5 years pre-diagnosis may be detrimental to one-year post diagnosis survival for ESCC (median RR = 1.73) although confidence intervals were wide (95% CI 0.83,4.17).

One benefit of the algorithm is that it does not add any additional information about individuals to the cancer registry data and so, unlike direct data linkage, does not exacerbate the issues of confidentiality and data security. (The imputed behaviours are only slightly more likely to be correct than an uninformed guess.) The algorithm also provides protection against biases. Data were obtained from the SEER cancer registries which are censuses with good population coverage. Many sampling and non-response biases in the BRFSS health behaviour data [41] are eliminated when using a census as the reference. However, we used rigid matching criteria and failed to match 20% of cases. Further investigation of the trade-off between exact matching and biases arising from failure to match is required.

As with direct data linkage, our investigations were limited to available health behaviour measures, rather than all clinically important risk factors. Potentially important health behaviours such as diet [11, 42] and hot beverages [42] were unavailable. The number and variety of auxiliary variables available for matching donor to recipient records was also limited. Our only investigation of clustering in health behaviours [43] was for the combination of current smoking and regular alcohol consumption.

The results display considerable uncertainty with few instances where the empirical confidence intervals excluded the null. The width of the confidence intervals is sensitive to *n*, *p*_*i*_ and *ρ*. Larger *n* can be achieved by looking at more common cancers, and/or combining data from more cancer registries and/or more years. The proportion with the health behaviour, *p*_*i*_, can be adjusted through inclusion and exclusion criteria (but will impact on *n*). Larger *ρ* requires more informative auxiliary variables for the imputation.

We do not have access to any true gold standard for validity testing. A gold standard would be an oesophageal cancer dataset where behaviour was measured 5 years prior to diagnosis.

## Conclusion

In this paper we have demonstrated a novel imputation-based algorithm for augmenting cancer registry data for epidemiological research and established its face-validity. The algorithm adds information obtained from an external data set with (presumed) no cases in common, to the cancer registry data via demographic variables in common. The algorithm is subject to much higher random error than direct data linkage (depending on how informative the demographic variables are), and requires larger sample sizes to compensate. However, it does avoid the aggravation of confidentiality issues (and associated data security costs) arising from direct data linkage.

We believe this algorithm is likely to allow, at least preliminary, investigations of a range of research questions which cannot be addressed through direct data linkage; due to insufficient individuals in common, insufficient matching variables and/or costs associated with data confidentiality and security. By increasing the range of research question which can be addressed with cancer registry data, the algorithm further augments the benefits of cancer registries.

## Supplementary information

**Additional file 1.** Provides a conceptual map of the steps in the imputation process.

**Additional file 2.** Charts the inclusion and exclusion of data records from both data sources.

**Additional file 3.** Shows the proportions of eligible SEER cancer cases that were unable to be matched with two donor records with non-missing smoking status.

**Additional file 4.** Details the mathematical model used to quantify the agreement between the pairs or imputed values assigned to each cancer case.

**Additional file 5.** Shows the strength of associations between candidate confounding variables and one-year survival. Shows why age group is an important potential confounder as both the proportion surviving and proportion with the health behaviour present decrease in older age groups.

**Additional file 6.** Shows the derivation of the mathematical relationship between the imputed relative risk and the true relative risk and thus introduces the formula used to correct for misclassification errors within the imputed health behaviours.

**Additional file 7.** Describes how health behaviour and survival status were assigned to cancer cases so as to produce the target relative risk in the simulated data sets.

**Additional file 8.** Tabulates the results of sub-group analyses on cancer stage I, II and III combined and for cancer stage IV.

## Data Availability

The SEER Research Data used in this study are made available to the public at no cost, subject to data-use agreement (https://seer.cancer.gov/data/). The BRFSS data sets used in this study are freely available from https://www.cdc.gov/brfss/index.html.

## Abbreviations

BRFSS: Behavioral Risk Factor Surveillance System
CI: Confidence interval
EAC: Esophageal adenocarcinoma
ESCC: Esophageal squamous cell carcinoma
HR: Hazard ratio
RR: Relative risk
SEER: Surveillance, Epidemiology, and End Results Program
