# Supplementary material for: Augmenting cancer registry data with health survey data with no cases in common: the relationship between pre-diagnosis health behaviour and post-diagnosis survival in oesophageal cancer
Source: BMC Cancer. 2020 Jun 1;20:496. doi: 10.1186/s12885-020-06990-3 (PMC7268470; doi:10.1186/s12885-020-06990-3)
Supplement: Supplementary file 1 — Additional file 1. Provides a conceptual map of the steps in the imputation process. [file 12885_2020_6990_MOESM1_ESM.docx]

Appendix A. The imputation algorithm

The conceptual steps of the imputation algorithm is summarised in the flow chart in Figure A.1. In practice, we operationalized this algorithm strata by strata rather than case by case. The imputation algorithm (and all analyses) was written in R software.

Figure A.1 A conceptual map of the algorithm used to impute the pre-diagnosis behaviour of oesophageal cancer cases.

All eligible SEER esophageal cancer data records

Select the next SEER cancer record

Locate all BRFSS behavior records which match^a^ the age group, gender, marital status, race, State of residence & year

Exclude SEER cancer case from analysis

Are there
at least 2 matching
BRFSS records?

No

Yes

Randomly select 2 of the matching BRFSS behavior records

Add the behavior of the BRFSS donor records to the SEER case

Exclude both BRFSS donor records from further matching

Are there
any remaining SEER
cases?

Yes

No

End

^a^ Matches are BRFSS health behaviour records which are 5 years earlier in time and one age-group younger than the corresponding SEER cancer case, with all other variables equal.
