# Supplementary material for: Augmenting cancer registry data with health survey data with no cases in common: the relationship between pre-diagnosis health behaviour and post-diagnosis survival in oesophageal cancer
Source: BMC Cancer. 2020 Jun 1;20:496. doi: 10.1186/s12885-020-06990-3 (PMC7268470; doi:10.1186/s12885-020-06990-3)
Supplement: Supplementary file 2 — Additional file 2. Charts the inclusion and exclusion of data records from both data sources. [file 12885_2020_6990_MOESM2_ESM.docx]

Appendix B. Inclusion and exclusion of SEER cancer cases and BRFSS health behaviour data records

Figure B.1. Flow chart of inclusions and exclusions of SEER oesophageal cancer cases

SEER oesophageal cancer cases from 2006 to 2014

*n*=35,084

Under 35 years of age

*n*=112

*n*=34,972

Missing survival time

*n*=456

*n*=34,516

Censored at <1-year post-diagnosis

*n*=301

*n*=34,215

Missing marital status and/or race

*n*=2,027

*n*=32,188

No BRFSS data records in same strata

*n*=580

*n*=31,608

Insufficient BRFSS records to provide two imputations

- Current smoker n=3,773
- Binge drinking n=3,858
- Heavy drinking n=3,859
- Physical activity n=3,778
- Obese n=3,812
- Current smoking with regular
  alcohol n=3,873

Included in the analysis

- Current smoking n=27,835
- Binge drinking n=27,750
- Heavy drinking n=27,749
- Physical activity n=27,830
- Obese n=27,796
- Current smoking with regular alcohol n=27,735

Figure B.2. Flow chart of inclusions and exclusions of BRFSS health behaviour data records

BRFSS data records from 2001 to 2009

*n*=3,018,830

Not from any of the 13 SEER States, *n*=2,182,086

*n*=836,744

Missing age, marital status and/or race, *n*=16,225

*n*=820,519

Cannot match^a^ age <30 *n*=89,229

*n*=731,290

Cannot match^a^ mixed race *n*=21,314

*n*=709,976

No SEER records in same strata, *n*=251,196

*n*=458,780

Missing behaviour data

- Current smoking n=2,531
- Binge drinking n=8,974
- Heavy drinking n=10,839
- Physical activity n=564
- Obese n=17,624
- Current smoking with regular alcohol n=12,894

Eligible donor records

- Current smoking n=456,249
- Binge drinking n=449,806
- Heavy drinking n=447,941
- Physical activity n=58,216
- Obese n=441,156
- Current smoking with regular alcohol n=445,886

Used in imputations

- Current smoker n=55,670 ● Physical activity n=55,660
- Binge drinking n=55,500 ● Obese n=55,592
- Heavy drinking n=55,498 ● Current smoker with regular alcohol n=55,470

^a^ All auxiliary variables could be coded identically in both SEER cancer registry data and BRFSS health behaviour data except the BRFSS data included an additional category for race. Unlike the SEER cancer registry data, the BRFSS data collection allowed respondents to describe their race as “mixed”. About 3.5% of BRFSS respondents selected this option. As these records did not match any SEER cancer registry records they did not contribute to the analysis. Similarly, with a minimum age of 35 years for SEER cancer cases and a 5-year lag, BRFSS respondents under 30 years of age could not match any SEER cancer registry records.
