# Supplementary material for: Augmenting cancer registry data with health survey data with no cases in common: the relationship between pre-diagnosis health behaviour and post-diagnosis survival in oesophageal cancer
Source: BMC Cancer. 2020 Jun 1;20:496. doi: 10.1186/s12885-020-06990-3 (PMC7268470; doi:10.1186/s12885-020-06990-3)
Supplement: Supplementary file 3 — Additional file 3. Shows the proportions of eligible SEER cancer cases that were unable to be matched with two donor records with non-missing smoking status. [file 12885_2020_6990_MOESM3_ESM.docx]

Appendix C. The availability of donor records for imputing the pre-diagnosis smoking status of eligible SEER oesophageal cancer cases.

Table C.1 shows the proportions of eligible SEER cancer cases that unable to be matched with two donor records with non-missing smoking status. Proportions of unmatched cases are shown for each category of each of the auxiliary variables used in matching. Results for the other behaviours were similar – varying slightly due to the different distribution of missing data in the different variables.

Results in Table C.1 show that failure to find donor records were more common:

- in earlier years when the number of people interviewed by BRFSS was smaller.
- in the older age categories where there are more cancer cases and relatively fewer BRFSS respondents.
- in California, where the population (and hence number of cancer cases) is quite large. The BRFSS surveys the same number per State regardless of population size.
- In marital and race groups where the BRFSS is known to be under-representative of the true population (1).

Table C.1 Number of SEER oesophageal cancer cases seeking donor records for current smoking behaviour and the proportion of these failing to obtain two donor records.

|  | Seeking Donor Records | |  | Failed to Obtain 2 Donor Records | |
| --- | --- | --- | --- | --- | --- |
|  | Frequency | % of total |  | Frequency | % of group |
| Total | 32,188 | 100.0% |  | 4,353 | 13.5% |
| Status at one year   - Dead - Alive | 17,490  14,698 | 54.3%  45.7% |  | 2,563  1,790 | 14.7%  12.2% |
| Cancer type   - ESCC - EAC | 10,454  17,950 | 32.5%  55.8% |  | 1,540  2,224 | 14.7%  12.4% |
| Year   - 2006 - 2007 - 2008 - 2009 - 2010 - 2011 - 2012 - 2013 - 2014 | 3,364  3,519  3,539  3,675  3,564  3,591  3,636  3,613  3,597 | 10.7%  10.9%  11.0%  11.4%  11.1%  11.2%  11.3%  11.2%  11.2% |  | 691  659  620  727  502  423  496  148  87 | 20.0%  18.7%  17.5%  19.8%  14.1%  11.8%  13.6%  4.1%  2.4% |
|  |  |  |  |  |  |

Table C.1 (continued). Number of SEER oesophageal cancer cases seeking donor records for current smoking behaviour and the proportion of these failing to obtain two donor records.

|  | Seeking Donor Records | |  | Failed to Obtain 2 Donor Records | |
| --- | --- | --- | --- | --- | --- |
|  | Frequency | % of total |  | Frequency | % of group |
| Total | 32,188 | 100.0% |  | 4,353 | 13.5% |
| Age group   - 35-39 - 40-44 - 45-49 - 50-54 - 55-59 - 60-64 - 65-69 - 70-74 - 75-79 - 80+ | 179  477  1,258  2,399  3,849  4,919  5,028  4,273  4,010  5,796 | 0.6%  1.5%  3.9%  7.5%  12.0%  15.3%  15.6%  13.3%  12.5%  18.0% |  | 2  5  25  138  368  692  810  725  736  852 | 1.1%  1.0%  2.0%  5.8%  9.6%  14.1%  16.1%  17.0%  18.4%  14.7% |
| Sex   - Male - Female | 25,131  7,057 | 78.1%  21.9% |  | 4,059  294 | 16.2%  4.2% |
| Marital status   - Married (including common law) - Divorced - Widowed - Single (Never married) | 18,363  4,046  4,394  5,384 | 57.0%  12.6%  13.7%  16.7% |  | 2238  287  518  1,309 | 12.2%  7.1%  11.8%  24.3% |
| Race   - White - Black - Asian or Pacific Islander - American Indian or Alaska Native | 27,184  3,410  1,406  187 | 84.5%  10.6%  4.4%  0.6% |  | 3,115  681  474  82 | 11.5%  20.0%  33.7%  43.9% |
| State of residence   - Alaska - California - Connecticut - Georgia - Hawaii - Iowa - Kentucky - Louisiana - Michigan - New Jersey - New Mexico - Utah - Washington | 39  11,672  1,799  3,599  479  1,727  3,028  1,972  1,902  3,629  648  613  1,980 | 0.1%  36.3%  5.6%  11.2%  1.5%  5.4%  9.4%  6.1%  5.9%  11.3%  2.0%  1.9%  6.2% |  | 39  3,551  34  308  55  35  53  81  70  81  9  10  26 | 100.0%  30.4%  1.9%  8.6%  11.5%  2.0%  1.8%  4.1%  3.7%  2.2%  1.4%  1.6%  1.3% |

References

1. Schneider KL, Clark MA, Rakowski W, Lapane KL. Evaluating the impact of non-response bias in the Behavioral Risk Factor Surveillance System (BRFSS). J Epidemiol Community Health. 2012;66(4):290-5.
