# Supplementary material for: Augmenting cancer registry data with health survey data with no cases in common: the relationship between pre-diagnosis health behaviour and post-diagnosis survival in oesophageal cancer
Source: BMC Cancer. 2020 Jun 1;20:496. doi: 10.1186/s12885-020-06990-3 (PMC7268470; doi:10.1186/s12885-020-06990-3)
Supplement: Supplementary file 4 — Additional file 4. Details the mathematical model used to quantify the agreement between the pairs or imputed values assigned to each cancer case. [file 12885_2020_6990_MOESM4_ESM.docx]

Appendix D. Calibration of the imputation algorithm

For each health behaviour we used the agreement between the two donor records to estimate of the amount of information on health behaviour retained by the cold deck imputation.

Let $p_{i}$ represent the proportion of imputed values where the behaviour is present. If the imputation process retained no information on behaviour, the expected proportion of behaviour present to behaviour present matches is $p_{i}^{2}$ - the agreement arising through chance alone. If the imputation process is informative, the proportion of behaviour present to behaviour present matches is greater than chance. Following Lunn & Davies (1) we modelled this improvement in matching as $p_{i}\left( 1-p_{i} \right) \rho$ where $\rho$ is a measure of correlation.

Table D.1 summarizes how the agreement between the two imputed values was modelled. The observed number of behaviour present to behaviour present matches is designed E and E is modelled as the number of behaviour present to behaviour present matches expected by chance alone, ${np}_{i}^{2}$, plus the excess matches arising from the information retained by the imputation algorithm, ${np}_{i}\left( 1-p_{i} \right)\rho$.

Table D.1 Observed and expected agreement between the two sets of imputations.

|  |  | Imputed behaviour #2 | | |
| --- | --- | --- | --- | --- |
|  |  | Behaviour present | Behaviour absent | total |
| Imputed behaviour #1 | Behaviour present  -observed  -expected | $E$  ${np}_{i}^{2}+{np}_{i}\left( 1-p_{i} \right)\rho$ | $F$  $np_{i}\left( 1-p_{i} \right)-{np}_{i}\left( 1-p_{i} \right)\rho$ | $E+F$  $np_{i}$ |
|  | Behaviour absent  -observed  -expected | $G$  $np_{i}\left( 1-p_{i} \right)-{np}_{i}\left( 1-p_{i} \right)\rho$ | $H$  $n\left( 1-p_{i} \right)^{2}+{np}_{i}\left( 1-p_{i} \right)\rho$ | $G+H$  $n\left( 1-p_{i} \right)$ |
|  | Total  -observed  -expected | $E+G$  $np_{i}$ | $F+H$  $n\left( 1-p_{i} \right)$ | $n$ |

The values $n, E, F, G$ and $H$ are obtained by cross tabulation of the two imputed values. Two potential estimates of $p_{i}$ are the observed proportion in the first set of imputed values and the observed proportion in the second set of imputed values. In the current study $p_{i}$ is estimated using the average of these two observed proportions:

$$\hat{p_{i}}=\frac{1}{2}\left( \frac{E+F}{n}+\frac{E+G}{n} \right)$$

The value of $\rho$ is estimated by the phi coefficient, $\varphi,$ (the correlation coefficient for dichotomous variables) between the pairs of imputed values.

References

1. Lunn AD, Davies SJ. A note on generating correlated binary variables. Biometrika. 1998;85(2):487-90.
