# Supplementary material for: Augmenting cancer registry data with health survey data with no cases in common: the relationship between pre-diagnosis health behaviour and post-diagnosis survival in oesophageal cancer
Source: BMC Cancer. 2020 Jun 1;20:496. doi: 10.1186/s12885-020-06990-3 (PMC7268470; doi:10.1186/s12885-020-06990-3)
Supplement: Supplementary file 5 — Additional file 5. Shows the strength of associations between candidate confounding variables and one-year survival. Shows why age group is an important potential confounder as both the proportion surviving and proportion with the health behaviour present decrease in older age groups. [file 12885_2020_6990_MOESM5_ESM.docx]

Appendix E. The association between health behaviours and cancer stage at diagnosis and survival time

The relationship between death within 1 year of diagnosis (yes/no) and each predictor was modelled separately using log-binary regression (sometimes called relative risk regression). The explanatory power of each predictor was summarised using the likelihood ratio test statistic and the area under the receiver operator curve. The likelihood ratio test statistics are only comparable if the degrees of freedom is equal. The area under the receiver operator curve is 0.5 for an uninformative predictor variable to 1.0 for a perfectly informative predictor.

Among the demographic variables, 5-year age group was the strongest predictor of dead within 1 year of diagnosis. Many of the behaviour variables also differ by age group. For example, the relationship between age group and died within 1 year of diagnosis is present in Figure E.1. To avoid confounding, the main analyses were age-adjusted.

Marital status, the next highest predictor, is strongly related to age. For example, 1.6% of those 35-39 year of age are widowed compared to 46.0% of those 80 or more years of age and 38.0% of those 35-39 years of age are single compared to 7.6% of those 80 or more years of age. Other demographic variables were found to be less predictive of death within one year of diagnosis.

As would be expected, cancer stage at diagnosis is a very strong predictor of death with one year of diagnosis with those diagnosed with stage I and II cancers having less than half the risk of dying as those diagnosed with stage IV disease.

Table E.1 Relationships between demographic variables and cancer stage at diagnosis and death within the first year of diagnosis.

|  | Estimated relative risk (RR) | Improvement over the null model  (likelihood ratio test statistic) | Area under the receiver operator curve |
| --- | --- | --- | --- |
| Year of diagnosis (n=34215)  - per year | 0.992 (0.988,0.995) | 18.8, df=1 | 0.514 (0.507,0.520) |
| Sex (n=34215)  - male  - female | reference  1.031 (1.007,1.054) | 6.8, df=1 | 0.506 (0.501,0.510) |
| Age group (n=34215)  - per 5 year age group | 1.060 (1.055,1.065) | 653.2, df=1 | 0.575 (0.569,0.581) |
| Marital status (n=32236)  - Married  - Divorced/separated  - Widowed  - Single | reference  1.171 (1.136,1.207)  1.341 (1.306,1.375)  1.207 (1.176,1.240) | 514.7, df=3 | 0.565 (0.559,0.570) |
| Race (n=34140)  - White  - Black  - Asian/Pacific  - American Native | reference  1.186 (1.154, 1.219)  1.062 (1.013,1.110)  1.122 (0.995,1.246) | 133.8, df=3 | 0.522 (0.518,0.526) |
| State of residence (n=34215)  - Alaska  - California  - Connecticut  - Georgia  - Hawaii  - Iowa  - Kentucky  - Louisiana  - Michigan  - New Jersey  - New Mexico  - Utah  - Washington | 0.832 (0.590, 1.081)  reference  0.857 (0.815,0.901)  1.034 (1.001,1.067)  1.120 (1.041,1.197)  0.900 (0.855,0.944)  1.031 (0.991,1.072)  1.044 (1.002,1.085)  0.939 (0.897,0.981)  0.915 (0.884,0.947)  1.020 (0.953,1.087)  0.975 (0.903,1.046)  0.956 (0.915,0.997) | 128.2, df=12 | 0.533 (0.527,0.539) |
|  |  |  |  |
| Cancer stage (n=34215)  - Stage I  - Stage II  - Stage III  - Stage IV  - Unknown | 0.462 (0.445,0.480)  0.464 (0.447,0.482)  0.597 (0.579,0.615)  reference  0.886 (0.866,0.905) | 4216.5, df=4 | 0.689 (0.684,0.695) |

df is an abbreviation of degrees of freedom

In Figure E.1 the association between age and one-year survival in SEER oesophageal cancer cases is depicted by the solid line and the relationships between age and health behaviours in the imputed data are shown. Notice that the proportion surviving one-year post diagnosis decreases in the older age groups as does the proportion with each health behaviour. As age is associated with both the predictor variable (health behaviour) and the outcome variable (death within one year of survival) it is likely to confound our understanding of the relationship between these variables.

Figure E.1 Proportion surviving and proportion with health behaviour by age group for SEER oesophageal cancer cases
