# Supplementary material for: Augmenting cancer registry data with health survey data with no cases in common: the relationship between pre-diagnosis health behaviour and post-diagnosis survival in oesophageal cancer
Source: BMC Cancer. 2020 Jun 1;20:496. doi: 10.1186/s12885-020-06990-3 (PMC7268470; doi:10.1186/s12885-020-06990-3)
Supplement: Supplementary file 6 — Additional file 6. Shows the derivation of the mathematical relationship between the imputed relative risk and the true relative risk and thus introduces the formula used to correct for misclassification errors within the imputed health behaviours. [file 12885_2020_6990_MOESM6_ESM.docx]

Appendix F. The relationship between the true relative risk and the imputed relative risk

In this section we derive the mathematical relationship between the imputed relative risk and the true relative risk. That is, we show how we correct for misclassification errors within the imputed health behaviours.

Let $p_{T}$ represent the proportion of cancer cases who have the behaviour and $p_{i}$ represent the proportion who are imputed to have that behaviour. If imputation were completely uninformative we would expect the number of the cancer cases with the behaviour who are correctly imputed to have the behaviour to be ${np}_{T}p_{i}$, just by chance alone. If imputation were informative we would expect more of the imputed values to be correct than by chance alone. This excess of correct matches can be modelled so long as estimates of $p_{T}$, $p_{i}$ and the correlation between them are available (1).

As the health behaviour of cancer patients is not recorded in the SEER cancer registry data, $p_{T}$ is unknown. One temptation may be to estimate $p_{T}$ based on the relative risk of incidence. For example, if current smoking is a risk factor for being diagnosed with oesophageal cancer with a relative risk of 2, then we could expect twice as many people with current smoking in the cancer population than in the general population and hence $p_{T}=2p_{i}$. The flaw here is that $p_{i}$ is not the smoking rate in the general population, it is the smoking rate in a sample with an identical demographic profile to the cancer cases.

For our analyses we have assumed that the proportion of imputed values with the behaviour provides a good approximation to the true proportion with the behaviour: $p_{T}=p_{i}$. This may or may not be true. (Suppose for example, even after correction for age, sex, race, marital status, State and year, that say education status remained an independent predictor of the prevalence of current smoking. Then any difference in education status between the SEER cancer registry cases and BRFSS health survey respondents would produce differences between $p_{T}$ and $p_{i}$.) We assume that the auxiliary variables which we have used are sufficient to encompass most of the variation in other factors which differ between the two data sets. (That is, we assume that differences between the two data sets in say education status are largely explained and corrected for by the existing auxiliary variables age, sex, race, marital status, State and year.)

Using $p_{i}$ as an estimate of $p_{T}$ and the correlation between the two sets of imputed values as an estimate of the correlation between the true behaviour and the imputed behaviour we can model the relationship between the true and imputed behaviour as shown in Table F.1.

Table F.1 A model of the association between the true and imputed health behaviours

|  |  | Imputed value of health behaviour | | |
| --- | --- | --- | --- | --- |
|  |  | Behaviour present | Behaviour absent | total |
| True (unknown) value of health behaviour | Behaviour present  - observed  - expected | $A$  $np_{i}^{2}+{np}_{i}\left( 1-p_{i} \right)\rho$ | $B$  $np_{i}\left( 1-p_{i} \right)- np_{i}\left( 1-p_{i} \right)\rho$ | $A+B$  $np_{T}$ |
|  | Behaviour absent  - observed  - expected | $C$  $np_{i}\left( 1-p_{i} \right)- np_{i}\left( 1-p_{i} \right)\rho$ | $D$  $n\left( 1-p_{i} \right)^{2}+ np_{i}\left( 1-p_{i} \right)\rho$ | $C+D$  $n\left( 1-p_{T} \right)$ |
|  | Total  - observed  - expected | $A+C$  $np_{i}$ | $B+D$  $n\left( 1-p_{i} \right)$ | $n$  $n$ |

Suppose the risk of death within one year of diagnosis is $r_{p}$ for cancer cases where the behaviour is present and $r_{a}$ for cancer cases where the behaviour is absent. The true relative risk of death within one year of diagnosis is ${RR}_{T}={r_{p}}/{r_{a}}.$ Notice that cells A and B in Table F.1 have $r_{p}$ risk of death and cells C and D have $r_{a}$ risk. Cross tabulating imputed health behaviour against one-year survival status would produce the results summarized in Table F.2.

Table F.2 Cross-tabulation of true one-year survival status against imputed health behaviour

|  |  | 12 month survival status | | |
| --- | --- | --- | --- | --- |
|  |  | died | lived | total |
| Imputed value of the health behaviour | Behaviour present  - observed  - expected | $I$  $Ar_{p}+$ $Cr_{a}$ | $J$  $A\left( 1-r_{p} \right)+$ $C\left( 1-r_{a} \right)$ | $I+J= A+C$  $np_{i}$ |
|  | Behaviour absent  - observed  - expected | $K$  $Br_{p}+Dr_{a}$ | $L$  $B\left( 1-r_{p} \right)+D\left( 1-r_{a} \right)$ | $K+L=B+D$  $n\left( 1-p_{i} \right)$ |
|  | Total  - observed  - expected | $I+K$ | $J+L$ | $n$  $n$ |

The relative risk calculated using the imputed data is

$${RR}_{i}= \frac{I/\left( I+J \right)}{K/\left( K+L \right)}$$

$$= \frac{I/{np_{i}}}{K/{n\left( 1-p_{i} \right)}}$$

$$=\frac{\left( Ar_{p} +Cr_{a} \right)/{np_{i}}}{\left( Br_{p} +Dr_{a} \right)/{n\left( 1-p_{i} \right)}}$$

$$=\frac{\left( Ar_{p} +Cr_{a} \right)}{np_{i}}\times\frac{n\left( 1-p_{i} \right)}{\left( Br_{p} +Dr_{a} \right)}$$

$$=\frac{\left( Ar_{p} +Cr_{a} \right)}{\left( Br_{p} +Dr_{a} \right)}\times\frac{\left( 1-p_{i} \right)}{p_{i}}$$

This equation can be re-arranged to produce the formula for the true relative risk as follows:

$${p_{i}Br_{p}RR}_{i}+{p_{i}Dr_{a}RR}_{i}=Ar_{p} +Cr_{a}-Ar_{p}p_{i}-Cr_{a}p_{i}$$

$$r_{p}\left( {p_{i}BRR}_{i}-A+Ap_{i} \right)= r_{a}\left( C-Cp_{i}-{p_{i}DRR}_{i} \right)$$

$${RR}_{T}= \frac{r_{p}}{r_{a}}= \frac{C-Cp_{i}-{p_{i}DRR}_{i}}{{p_{i}BRR}_{i}-A+Ap_{i}}$$

Now substituting the models for $A, B, C$ and $D$ shown in Table F.1:

$${RR}_{T}= \frac{n\left( p_{i}\left( 1-p_{i} \right)- p_{i}\left( 1-p_{i} \right)\rho\right)-n\left( \left( p_{i}\left( 1-p_{i} \right)- p_{i}\left( 1-p_{i} \right)\rho\right) \right)p_{i}-{p_{i}\left( n\left( \left( 1-p_{i} \right)\left( 1-p_{i} \right)+ p_{i}\left( 1-p_{i} \right)\rho\right) \right)RR}_{i}}{{p_{i}\left( n\left( p_{i}\left( 1-p_{i} \right)- p_{i}\left( 1-p_{i} \right)\rho\right) \right)RR}_{i}-n\left( p_{i}p_{i}+p_{i}\left( 1-p_{i} \right)\rho\right)+\left( n\left( p_{i}p_{i}+p_{i}\left( 1-p_{i} \right)\rho\right) \right)p_{i}}$$

Note $n$ and $p_{i}$cancel out to give:

$${RR}_{T}= \frac{\left( 1-p_{i} \right)- \left( 1-p_{i} \right)\rho-\left( p_{i}\left( 1-p_{i} \right)- p_{i}\left( 1-p_{i} \right)\rho\right)-{\left( \left( 1-p_{i} \right)\left( 1-p_{i} \right)+ p_{i}\left( 1-p_{i} \right)\rho\right)RR}_{i}}{{\left( p_{i}\left( 1-p_{i} \right)- p_{i}\left( 1-p_{i} \right)\rho\right)RR}_{i}-\left( p_{i}+\left( 1-p_{i} \right)\rho\right)+p_{i}p_{i}+p_{i}\left( 1-p_{i} \right)\rho}$$

$$= \frac{1-p_{i}- \left( \rho-p_{i}\rho\right)-\left( p_{i}-p_{i}^{2}- \left( p_{i}\rho-p_{i}^{2}\rho\right) \right)-{\left( 1-p_{i}-p_{i}+p_{i}^{2}+ \left( p_{i}-p_{i}^{2} \right)\rho\right)RR}_{i}}{{\left( p_{i}-p_{i}^{2}- \left( p_{i}-p_{i}^{2} \right)\rho\right)RR}_{i}-\left( p_{i}+\rho-p_{i}\rho\right)+p_{i}p_{i}+\left( p_{i}-p_{i}^{2} \right)\rho}$$

$$= \frac{1-p_{i}- \rho+p_{i}\rho-\left( p_{i}-p_{i}^{2}- p_{i}\rho+p_{i}^{2}\rho\right)-{\left( 1-p_{i}-p_{i}+p_{i}^{2}+ p_{i}\rho-p_{i}^{2}\rho\right)RR}_{i}}{{\left( p_{i}-p_{i}^{2}- p_{i}\rho+p_{i}^{2}\rho\right)RR}_{i}-p_{i}-\rho+p_{i}\rho+p_{i}p_{i}+p_{i}\rho-p_{i}^{2}\rho}$$

$$= \frac{1-p_{i}- \rho+p_{i} \rho-p_{i}+p_{i}^{2}+ p_{i} \rho-p_{i}^{2} \rho-\left( {RR}_{i}-p_{i}{RR}_{i}-p_{i}{RR}_{i}+p_{i}^{2}{RR}_{i}+ p_{i} \rho{RR}_{i}-p_{i}^{2} \rho{RR}_{i} \right)}{p_{i}{RR}_{i}-p_{i}^{2}{RR}_{i}- p_{i} \rho{RR}_{i}+p_{i}^{2} \rho{RR}_{i}-p_{i}- \rho+p_{i} \rho+p_{i}p_{i}+p_{i} \rho-p_{i}^{2} \rho}$$

$$= \frac{1-p_{i}- \rho+p_{i} \rho-p_{i}+p_{i}^{2}+ p_{i} \rho-p_{i}^{2} \rho-{RR}_{i}+p_{i}{RR}_{i}+p_{i}{RR}_{i}-p_{i}^{2}{RR}_{i}- p_{i} \rho{RR}_{i}+p_{i}^{2} \rho{RR}_{i}}{p_{i}{RR}_{i}-p_{i}^{2}{RR}_{i}- p_{i} \rho{RR}_{i}+p_{i}^{2} \rho{RR}_{i}-p_{i}- \rho+p_{i} \rho+p_{i}^{2}+p_{i} \rho-p_{i}^{2} \rho}$$

$$= \frac{-{RR}_{i}+1+p_{i}{RR}_{i}-p_{i}- \rho+p_{i}\rho+p_{i}{RR}_{i}-p_{i}-p_{i}^{2}{RR}_{i}+p_{i}^{2}- p_{i}\rho{RR}_{i}+ p_{i}\rho+p_{i}^{2}\rho{RR}_{i}-p_{i}^{2}\rho}{p_{i}{RR}_{i}-p_{i}-p_{i}^{2}{RR}_{i}+p_{i}^{2}- p_{i}\rho{RR}_{i}+p_{i}\rho+p_{i}^{2}\rho RR-p_{i}^{2}\rho-\rho+p_{i}\rho}$$

$$= \frac{-\left( {RR}_{i}-1 \right)+p_{i}\left( {RR}_{i}-1 \right)- \rho\left( 1-p_{i} \right)+p_{i}\left( {RR}_{i}-1 \right)-p_{i}^{2}\left( {RR}_{i}-1 \right)- p_{i}\rho\left( {RR}_{i}- 1 \right)+p_{i}^{2}\rho\left( {RR}_{i}-1 \right)}{p_{i}\left( {RR}_{i}-1 \right)-p_{i}^{2}\left( {RR}_{i}-1 \right)- p_{i}\rho\left( {RR}_{i}-1 \right)+p_{i}^{2}\rho\left( RR-1 \right)-\rho\left( 1-p_{i} \right)}$$

$$= \frac{-\left( {RR}_{i}-1 \right)+\left( {RR}_{i}-1 \right)\left( p_{i}-p_{i}^{2}- p_{i}\rho+p_{i}^{2}\rho\right)- \rho\left( {1-p}_{i} \right)+p_{i}\left( {RR}_{i}-1 \right)}{\left( {RR}_{i}-1 \right)\left( p_{i}-p_{i}^{2}- p_{i}\rho+p_{i}^{2}\rho\right)-\rho\left( 1-p_{i} \right)}$$

$${RR}_{T}= \frac{\left( {RR}_{i}-1 \right)\left( p_{i}-p_{i}^{2}- p_{i}\rho+p_{i}^{2}\rho\right)- \rho\left( {1-p}_{i} \right)-\left( 1-p_{i} \right)\left( {RR}_{i}-1 \right)}{\left( {RR}_{i}-1 \right)\left( p_{i}-p_{i}^{2}- p_{i}\rho+p_{i}^{2}\rho\right)-\rho\left( 1-p_{i} \right)}$$

$$= 1-\frac{\left( 1-p_{i} \right)\left( {RR}_{i}-1 \right)}{\left( {RR}_{i}-1 \right)\left( p_{i}-p_{i}^{2}- p_{i}\rho+p_{i}^{2}\rho\right)-\rho\left( 1-p_{i} \right)}$$

$$= 1-\frac{\left( 1-p_{i} \right)\left( {RR}_{i}-1 \right)}{\left( {RR}_{i}-1 \right)p_{i}\left( 1-p_{i}- \rho+p_{i}\rho\right)-\rho\left( 1-p_{i} \right)}$$

$$= 1-\frac{\left( 1-p_{i} \right)\left( {RR}_{i}-1 \right)}{\left( {RR}_{i}-1 \right)p_{i}\left( 1-p_{i} \right)\left( 1-\rho\right)-\rho\left( 1-p_{i} \right)}$$

$$= 1-\frac{\left( {RR}_{i}-1 \right)}{\left( {RR}_{i}-1 \right)p_{i}\left( 1-\rho\right)-\rho}$$

Notice that when ${RR}_{i}=1$ then ${RR}_{T}= 1$ as we would expect. However, extreme values of $p_{i}$ and/or $\rho$ may be problematic. When $\rho=0$ then ${RR}_{T}= 1-\frac{1}{p_{i}}$ which is always negative and hence an impossible value for a relative risk. Also when either $p_{i}$ or $\left( {1-p}_{i} \right)$ approach 0, ${RR}_{T}$ approaches$1-\frac{\left( {RR}_{i}-1 \right)}{0-\rho}$, or $\left( {RR}_{T}-1 \right)$ approaches $\frac{1}{\rho}\left( {RR}_{i}-1 \right)$. That is when $p_{i}$ or $\left( {1-p}_{i} \right)$ are close to zero, ${RR}_{T}$ becomes particularly sensitive to small $\rho$.

References

1. Oman SD, Zucker DM. Modelling and generating correlated binary variables. Biometrika. 2001;88(1):287-90.
