# Supplementary material for: Augmenting cancer registry data with health survey data with no cases in common: the relationship between pre-diagnosis health behaviour and post-diagnosis survival in oesophageal cancer
Source: BMC Cancer. 2020 Jun 1;20:496. doi: 10.1186/s12885-020-06990-3 (PMC7268470; doi:10.1186/s12885-020-06990-3)
Supplement: Supplementary file 7 — Additional file 7. Describes how health behaviour and survival status were assigned to cancer cases so as to produce the target relative risk in the simulated data sets. [file 12885_2020_6990_MOESM7_ESM.docx]

Appendix H. Simulating data with prescribed relative risks

We sought to assign health behaviour and survival status to cancer cases in such a way as to produce the target relative risk ${RR}_{t}$ while maintaining the proportion of people with the behaviour and proportion of people dying within one year at the true levels. We assumed the first set of imputed values were actually the true measurements of behaviour. This ensured the correct proportion of people with the behaviour. We next simulated survival status for these behaviours to deliver the target relative risk.

In Table G.1 below:

- $A+B$ is the observed number of cancer cases who have the behaviour;
- $C+D$ is the observed number of cancer cases who do not have the behaviour;
- $A+C$ is the observed number of cancer cases who died within 12 months;
- $B+D$ is the observed number of cancer cases who lived for 12 months or more; and
- $a'$ and $c'$ be the number of deaths required to produce a target relative risk, ${RR}_{t}$

Table G.1 Values required for the simulation

|  |  | 12 month survival status | | |
| --- | --- | --- | --- | --- |
|  |  | died | lived | total |
| Imputed value of the health behaviour | Behaviour present | $a'$ |  | $A+B$ |
|  | Behaviour absent | $c'$ |  | $C+D$ |
|  | Total | $A+C$ | $B+D$ | $n$ |

To achieve any given target relative risk ${RR}_{t}$ we need to select $a'$ and $c'$ such that

$$\frac{{a'}/\left( A+B \right)}{{c'}/\left( C+D \right)}={RR}_{t}$$

First, note that

$$a^{'}+c^{'}=A+C$$

So we can replace $a^{'}$ by

$a^{'}=\left( A+C \right)-c^{'}$

To get:

$$\frac{\left( \left( A+C \right)-c^{'} \right)/\left( A+B \right)}{{c'}/\left( C+D \right)}={RR}_{t}$$

Solving for $c^{'}$ :

$$\frac{\left( A+C \right)-c^{'}}{A+B}=\frac{{RR}_{t}c'}{C+D}$$

$$\left( A+C \right)\left( C+D \right)-c^{'}\left( C+D \right)={RR}_{t}c'\left( A+B \right)$$

$$\left( A+C \right)\left( C+D \right)={RR}_{t}c^{'}\left( A+B \right)+c^{'}\left( C+D \right)$$

$$\left( A+C \right)\left( C+D \right)=c^{'}\left( {RR}_{t}\left( A+B \right)+\left( C+D \right) \right)$$

$$c^{'}=\frac{\left( A+C \right)\left( C+D \right)}{{RR}_{t}\left( A+B \right)+\left( C+D \right)}$$

Which, it turn, allows the value to be calculated for:

$$a^{'}=\left( A+C \right)-c^{'}$$

For data records in the behaviour present group we randomly assign 12 month survival status with probability of dying within 12 months equal to ${a^{'}}/\left( A+B \right)$. For those imputed to be in the behaviour absent group we randomly assign probability of dying within 12 months equal to ${c^{'}}/\left( C+D \right)$.

We implemented these random selections using the sample() command in R software.
