# Supplementary material for: Augmenting cancer registry data with health survey data with no cases in common: the relationship between pre-diagnosis health behaviour and post-diagnosis survival in oesophageal cancer
Source: BMC Cancer. 2020 Jun 1;20:496. doi: 10.1186/s12885-020-06990-3 (PMC7268470; doi:10.1186/s12885-020-06990-3)
Supplement: Supplementary file 8 — Additional file 8. Tabulates the results of sub-group analyses on cancer stage I, II and III combined and for cancer stage IV. [file 12885_2020_6990_MOESM8_ESM.docx]

Appendix I. Subgroup analyses for cancer stage at diagnosis

Table I.1 follows the same format as Table 3 in the main document. The final column shows age-adjusted estimates of the relative risk for each health behaviour for all cases, individuals with stage IV (metastatic) cancer at diagnosis and for stages I-III cancers at diagnosis combined. Results are available for current smoking, non-work related physical activity and obesity each 5 years prior to diagnosis. Review of the relative risks suggests that these pre-diagnosis health behaviours appear to have larger effects on survival for those with stage I-III cancer at diagnosis than for those with metastatic cancers.

Table I.1. Estimated relative risks of 1-year survival derived from imputed pre-diagnosis behaviours for SEER oesophageal cancer cases, 2006-2014; unadjusted and age adjusted. Showing subgroup analysis for cancer stage at diagnosis.

|  | Imputed RR  $\left( {RR}_{i} \right)$ | | Impossible Result $\left( {RR}_{i}<0 \right)$ | Estimated True RR  $\left( {RR}_{T} \right)$ | | |  | | Age-adjusted Imputed RR  $\left( {adjRR}_{i} \right)$ | | | Impossible  Result $\left( {adjRR}_{i}<0 \right)$ | | | Age-adjusted Estimated True RR  $\left( adj{RR}_{T} \right)$ | | |
| --- | --- | --- | --- | --- | --- | --- | --- | --- | --- | --- | --- | --- | --- | --- | --- | --- | --- |
|  | Median | 95% CI | Frequency | Median | 95% CI | |  | | Median | 95% CI | | Frequency | | | Median | 95% CI | |
| Current smoking  All  Stage IV  Stage I, II or III | 0.986  0.996  0.969 | 0.954,1.009  0.968,1.024  0.917,1.120 | 0  0  3 | 0.806  0.949  0.570 | 0.380,1.130  0.531,1.455  0.125,1.186 ^b^ | |  | | 1.051  1.033  1.085 | 1.014,1.078 ^a^  1.000,1.063 ^a^  1.025,1.138 ^a^ | | 0  0  0 | | | 1.794  1.486  2.499 | 1.215,2.357^a^  1.006,2.360 ^a^  1.428,3.900 ^a^ | |
| Binge drinking  All  Stage IV  Stage I, II or III | 0.933  0.965  0.866 | 0.900,0.964 ^a^  0.933,0.997 ^a^  0.803,0.928 ^a^ | 49  12  97 | failed  failed  failed | failed  failed  failed | |  | | 0.997  1.003  0.977 | 0.961,1.032  0.967,1.137  0.906,1.052 | | 1  0  18 | | | 0.951  1.050  failed | 0.445,1.539 ^b^  0.409,1.833  failed | |
| Heavy drinking  All  Stage IV  Stage I, II or III | 0.981  0.993  0.955 | 0.932,1.028  0.936,1.041  0.871,1.052 | 61  49  72 | failed  failed  failed | failed  failed  failed | |  | | 1.010  1.006  1.006 | 0.963,1.060  0.948,1.058  0.912,1.101 | | 23  31  39 | | | failed  failed  failed | failed  failed  failed | |
| Physical activity  All  Stage IV  Stage I, II or III | 0.954  0.976  0.915 | 0.934,0.978 ^a^  0.946,0.999 ^a^  0.868,0.947 ^a^ | 0  1  17 | 0.319  0.541  failed | 0.165,0.564 ^a^  0.207,0.994 ^a,b^  failed | |  | | 0.974  0.987  0.947 | 0.956,1.001  0.958,1.008  0.897,0.978 ^a^ | | 0  0  3 | | | 0.507  0.711  0.258 | 0.307,1.030  0.306,1.474^b^  0.039,0.588 ^a^ | |
| Obese  All  Stage IV  Stage I, II or III | 0.969  0.988  0.954 | 0.946,0.993 ^a^  0.963,1.015  0.912,1.002 | 24  8  54 | failed  failed  failed | failed  failed  failed |  | | 1.008  1.008  1.018 | | | 0.983,1.036  0.981,1.034  0.974,1.067 | | 0  3  2 | 1.262  1.267  1.794 | | | 0.559,2.931  0.506,4.319 ^b^  0.376,8.612 ^b^ |
| Current smoking with regular drinking  All  Stage IV  Stage I, II or III | 0.987  0.983  0.967 | 0.930,1.058  0.936,1.040  0.860,1.097 | 40  43  59 | failed  failed  failed | failed  failed  failed |  | | 1.044  1.016  1.073 | | | 0.986,1.120  0.966,1.071  0.956,1.228 | | 2  22  9 | 3.254  failed  failed | | | 0.771,11.843  failed  failed |

Imputed RR $\left( {RR}_{i} \right)$ – the relative risk calculated using the imputed behaviour

Impossible result – instances where the estimated true relative risk was impossible (a negative value)

Estimated True RR $\left( {RR}_{T} \right)$ – the estimated true relative risk derived from the imputed relative risk and calibration parameters $\hat{p_{i}}$ and $\hat{\rho}$

Median= median of 100 repetitions of the imputation algorithm,

95% CI= empirical 95% confidence interval created from the 2.5 and 97.5 percentiles obtained from 100 repetitions of the imputation algorithm,

^a^ 95% confidence intervals exclude no association (i.e. exclude relative risk equals 1)

^b^ excludes impossible result
